# Compressive force-induced succinate production via metabolic reprogramming in periodontal ligament cells promotes orthodontic tooth movement

**DOI:** 10.1186/s40510-025-00563-5

**Published:** 2025-05-19

**Authors:** Jiahong Shi, Lulu Wang, Liliang Shan, Meng Zhu, Yu Chen, Houxuan Li, Lang Lei

**Affiliations:** 1 Department of Periodontics, Nanjing Stomatological Hospital, Affiliated Hospital of Medical School, Institute of Stomatology, Nanjing University, Nanjing, China; 2 Present Address: Department of Orthodontics, Nanjing Stomatological Hospital, Affiliated Hospital of Medical School, Institute of Stomatology, Nanjing University, Nanjing, China; 3 Department of Orthodontics, Stomatology Hospital of General Hospital, Ningxia Medical University, Yinchuan, Ningxia Hui Autonomous Region, China; 4 Department of Dentistry, People’s Hospital of Longhua, Shenzheng, China

**Keywords:** Gingival crevicular fluid, Orthodontic tooth movement, Succinate, Succinate receptor, Bone remodeling

## Abstract

**Objective:**

This study aimed to elucidate metabolic alterations in gingival crevicular fluid (GCF) during orthodontic tooth movement (OTM) and investigate the role of the succinate-SUCNR1 axis in bone resorption and tooth movement.

**Results:**

OTM was accompanied by the change of TCA cycle and increase of succinate in the human GCF. Succinate accumulation was observed in periodontal ligament cells (PDLCs) under compressive force, accompanied by increase of glycolysis and decrease of succinic dehydrogenase activity. Suppression of the succinate-SUCNR1 axis reduced osteoclastogenesis in BMDMs. OTM slowed down in the SUCNR1^−^/^−^ mice when compared with wild mice.

**Conclusion:**

OTM is accompanied by the increase of succinate in periodontal tissues. Compressive force induces metabolic reprogramming in PDLCs, leading to enhanced succinate production. Succinate promotes macrophage migration and osteoclast differentiation via the SUCNR1 axis, ultimately facilitating orthodontic tooth movement. These findings provide a new potential therapeutic target for regulating periodontal tissue remodeling during orthodontic treatment.

## Introduction

Orthodontic tooth movement (OTM), the process of straightening misaligned teeth to a proper position after application of mechanical force, is accompanied by the remodeling of periodontal tissues. Understanding the underlying mechanism of OTM is essential for achieving an optimal outcome with minimal tissue damage. As the direct sensing cells of orthodontic force, periodontal ligament cells (PDLCs) exhibit high sensitivity to mechanical stimulation [1]. Moreover, PDLCs are thought to serve as a bridge between local tissues and the innate immune system through the secretion of chemical mediators [2]. However, the precise mechanisms underlying the conversion of mechanical stimulation into biological signals by PDLCs remain elusive and warrant further investigation.

Orthodontic force, especially on the pressure side, may induce microvascular obstruction and create a localized hypoxic milieu in the periodontal ligament microenvironment, ultimately leading to the occurrence of aseptic inflammation [3]. In response to applied force, chemical mediators such as growth factors, neurotransmitters and colony-stimulating factors were discharged from resident periodontal ligament cells (PDLCs) and recruited defense cells from blood vessels [4, 5]. Such sterile inflammatory response ultimately results in OTM to relieve the mechanical strain on the periodontal tissue [6]. It has been demonstrated that inflammatory mediators may be released into the gingival crevicular sulcus [4, 7] and GCF can serve as a source for detecting potential biomarkers of OTM [8]. It is known that the interaction between PDLCs and macrophages is likely to modulate the osteoclast differentiation and facilitate OTM [9]. These mediators in GCF may subsequently trigger processes that promote the migration and differentiation of monocytes/macrophages.

The metabolic transition from mitochondrial ATP synthesis to aerobic glycolysis constitutes a critical adaptive mechanism under inflammatory or hypoxic conditions [10]. Increased aerobic glycolysis was indicated by the upregulated mRNA expression of genes associated with several crucial rate-limiting enzymes, such as hexokinase 2 (HK2), 6-phosphofructo-2-kinase (PFKFB3), pyruvate kinase (PKM) and lactate dehydrogenase A (LDHA) [11]. Meanwhile, succinate dehydrogenase (SDH), consisting of subunits such as SDHa and SDHb, plays a critical role in converting succinate into fumarate [12]. Impaired SDH activity occurs under conditions of inadequate oxygen supply, further contributing to succinate accumulation in the mitochondrial matrix [13]. Succinate, traditionally considered as an intermediate in the tricarboxylic acid (TCA) cycle, has been recognized as a key mediator in inflammatory processes [14]. Elevated intracellular succinate stimulates the activation of hypoxia-inducible factor-1α (HIF-1α), a critical transcription factor that controls cellular adaptations to hypoxia, enhancing the transcription of pro-inflammatory genes [15]. Activation of HIF-1α dampens the activity of SDH, leading to pileup of succinate in the mitochondria [16]. Succinate receptor 1 (SUCNR1), one of G-protein-coupled receptor (GPCR), is a specific receptor to succinate. As an extracellular signaling molecule, succinate activates SUCNR1 in both autocrine and paracrine modes, forming the succinate-SUCNR1 signaling axis [17]. Elevated succinate may serve as a mediator promoting the progression of atherosclerotic lesions, gastrointestinal disorders and diabetes mellitus via the SUCNR1 pathway [18–20]. However, the function of succinate-SUCNR1 in OTM has never been reported and needs to be further investigated.

In this study, metabolomics analysis of GCF during OTM revealed increased succinate levels and alterations in the TCA cycle. Under compressive force loading, the metabolism of PDLCs is rewired from oxidative phosphorylation to glycolysis, which is accompanied with succinate accumulation. Suppression of succinate-SUCNR1 axis reduced the osteoclastogenesis in BMDMs and the distance of OTM in the animal model. Collectively, these findings suggest that compressive force induces metabolic reprogramming in PDLCs to enhance succinate production, which promotes macrophage migration and osteoclast differentiation via the SUCNR1 axis, ultimately facilitating tooth movement.

## Materials and methods

### Collection and processing of clinical specimen

#### Ethics Approval, Patient Recruitment and Inclusion Criteria

This study was approved by the Institutional Review Board of (NJSH-2023NL-036). A total of 13 patients referred to the Department of Orthodontics, between July 2022 and July 2023 were included in this study. All patients were informed of the study’s aim and provided consent before enrollment.

The inclusion criteria for this study were: (1) aged 18–30 years; (2) Angle Class I malocclusion with moderate crowding (5–8 mm) in the mandibular anterior segment; (3) planned extraction of four first premolars as part of the orthodontic treatment; and (4) simultaneous placement of fixed orthodontic appliances in both the upper and lower arches.

The exclusion criteria included: (1) chronic periodontitis; (2) systemic diseases; (3) prior orthodontic treatment; (4) use of medications that may affect periodontal status within the past 3 months; (5) generalized caries; (6) pregnant or nursing; (7) congenital loss of permanent teeth excluding third molars.

#### Orthodontic appliance placement

The conventional four-winged brackets (0.022 × 0.028-inch slot) (American Orthodontics, US) were used for all patients in this study. Initial archwire, 0.012-inch super-elastic Nickel-titanium (NiTi) (IMD, China), were inserted to align the dentition during the first month.

#### Clinical index measurements

Clinical index measurements of mandibular anterior teeth in all subjects were conducted at different timepoints, and all indices were examined by the same clinician. The indices included Probing Depth (PD), Clinical Attachment Level (CAL), Gingival Index (GI) and Plaque Index (PLI). PD and PLI were measured as previously described [21, 22]. Based on the color of the gums and probing results, the GI was classified as 4 levels: 0 = healthy and normal soft tissue; 1 = slightly inflammation without bleeding on probing; 2 = gingival edema with bleeding on probing; 3 = spontaneous bleeding or gum ulceration.

#### GCF collection and metabolite profiling by gas chromatography–mass spectrometry (GC–MS)

**Fig. 1:**
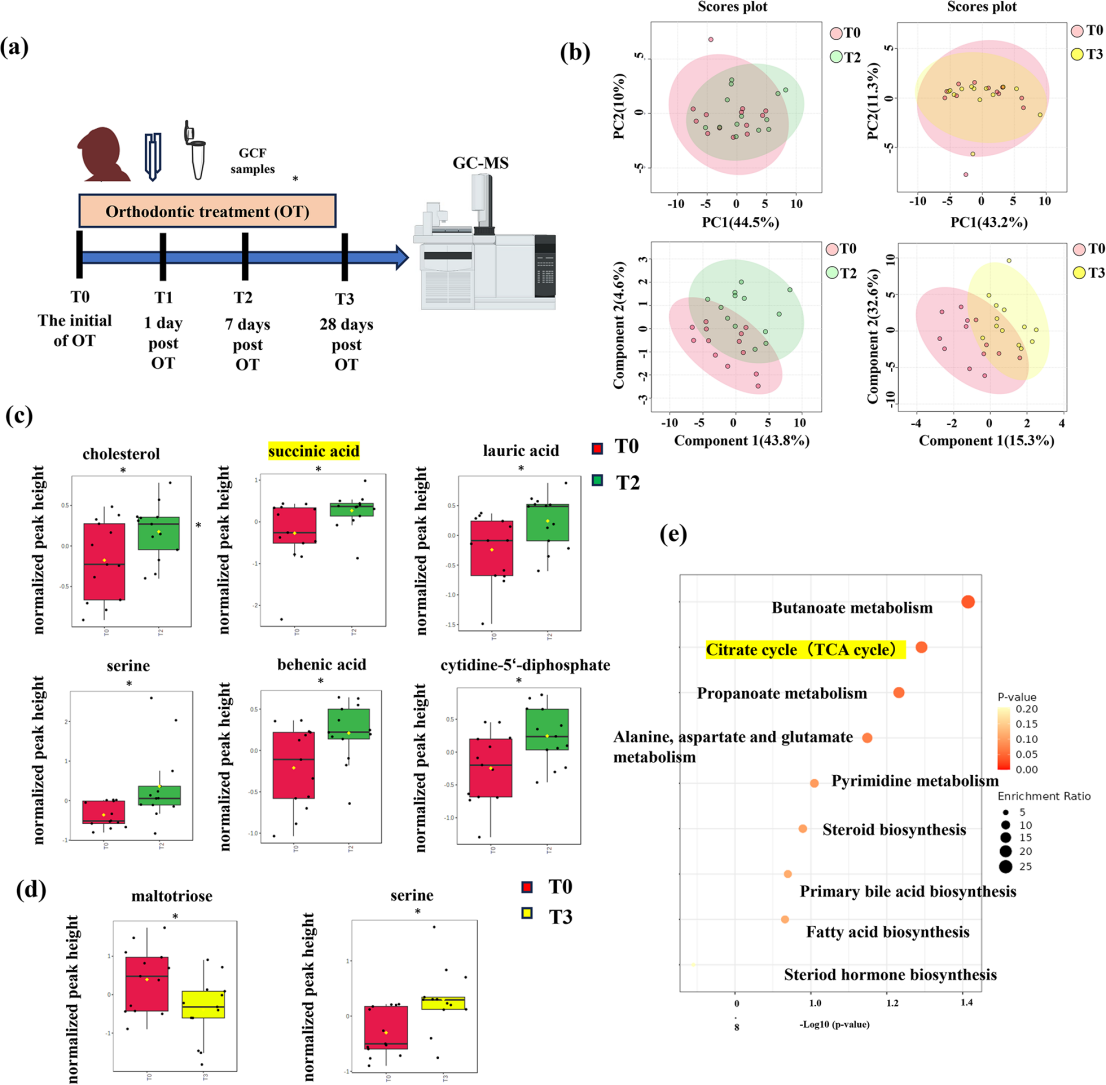
Mechanical force promoted succinate production in GCF during the progress of OTM. **a** Sequence of treatment and examinations (T0, T1, T2 and T3). **b** Principal Component Analysis (PCA) and Partial least squares Discriminant Analysis (PLS- DA) of GCF between T0-T2 and T0-T3. The results illuminated the difference between sample groups. **c**, **d** Metabolites showing significant differences with a *p* value < 0.05 according to *t*-test between T0-T2 (**c**) and T0-T3 (**d**). (*p < 0.05) **e** Overview of enriched metabolite sets analysis between T0 and T2. The bubble size indicates the enrichment ratio of the pathway (the larger the size is, the larger is the enrichment ratio); the Y-axis and bubble color indicate the *p* value in enrichment analysis (the darker the color is, the smaller is the *p* value)

The sequence of treatment and examinations (T0, T1, T2 and T3) was shown in Fig. 1a. GCF samples were collected from the mesio-buccal (MB) and disto-buccal (DB) sites of the six mandibular anterior teeth located between the mandibular canines. 12 Perio-Papers were collected at 4 timepoints: before first application of orthodontic forces (T0), 1 day after (T1), 7 days after (T2), and 28 days after (T3) from each subject. Strips contaminated with blood were discarded. The strips were immediately sealed in 1.5-mL Eppendorf (EP) tubes and stored at − 80 °C until further use for metabolic assessment.

The strips placed in EP tubes were extracted with 500 µL ice -cold methanol containing an internal standard (12.5 µg/mL 1,2–13^ C^ Myristic acid). Samples were soaked for 10 min, vortexed for 10 min, and then centrifuged at 4 ℃ and 18,000 rpm for 10 min. A 300 µL aliquot of the sample was taken and the supernatant was dried without heating. Subsequently, 30 µL of methoxypyridine (10 mg/mL) was added to the sample after evaporation. The sample was vortexed for 5 min at 30 ℃ and oscillated at 300 rpm for 1.5 h. Next, 30 µL of N, O-Bis (trimethylsilyl) trifluoroacetamide (BSTFA) was added. The sample was vortexed at 37℃ and 300 rpm for 1 min and then oscillated for 0.5 h. After the derivatization reaction was completed, the sample was centrifuged at 4℃ and 18,000 rpm for 10 min, and the supernatant (50 µL) was collected as the final sample.

A gas chromatograph system (Trace 1310-TSQ 8000 Evo, Thermo Fisher, San Jose, CA, USA), equipped with a chromatographic column (TG-5MS capillary column, 30 m × 250 μm inner diameter, 0.25 μm film thickness, Thermo Fisher, San Jose, CA, USA), was used for the separation of derivatives. The collision energy of 70 eV was applied. The injection temperature was stabilized at 280 °C, and splitless injection was performed using helium as the carrier gas with an injection volume of 1 µL. In the full-scan mode, the mass scan was set from 50 to 500 m/z.

### Cellular assays and analyses

#### Cell culture

Teeth were acquired from healthy premolars of patients (aged 10–18 years) for orthodontic treatment. Informed consent was obtained from each participant. PDLCs were cultured as previously described [23].

Bone marrow-derived macrophages (BMDMs) from SUCNR1-/- or wild-type (WT) mice were isolated and cultured with 30 ng/ml macrophage colony-stimulating factor (M-CSF) and 50 ng/ml receptor activator of nuclear factor kappa-B ligand (RANKL) for 3–7 days to induce osteoclast differentiation, as previously reported [24].

To investigate the role of succinate in PDLCs or BMDMs, exogenous succinate (sodium succinate dibasic hexahydrate, S9637, Sigma-Aldrich, USA) was supplemented into the culture medium at varying concentrations (1 mM or 5 mM). To inhibit the role of SUCNR1 in BMDMs, the SUCNR1-antagonist 4c (Wuhe BioTech, Liaoning, China) was added to the culture medium at a concentration of 10 µM. The same dosage of DMSO was added to the control group.

#### Application of compressive force

**Fig. 2:**
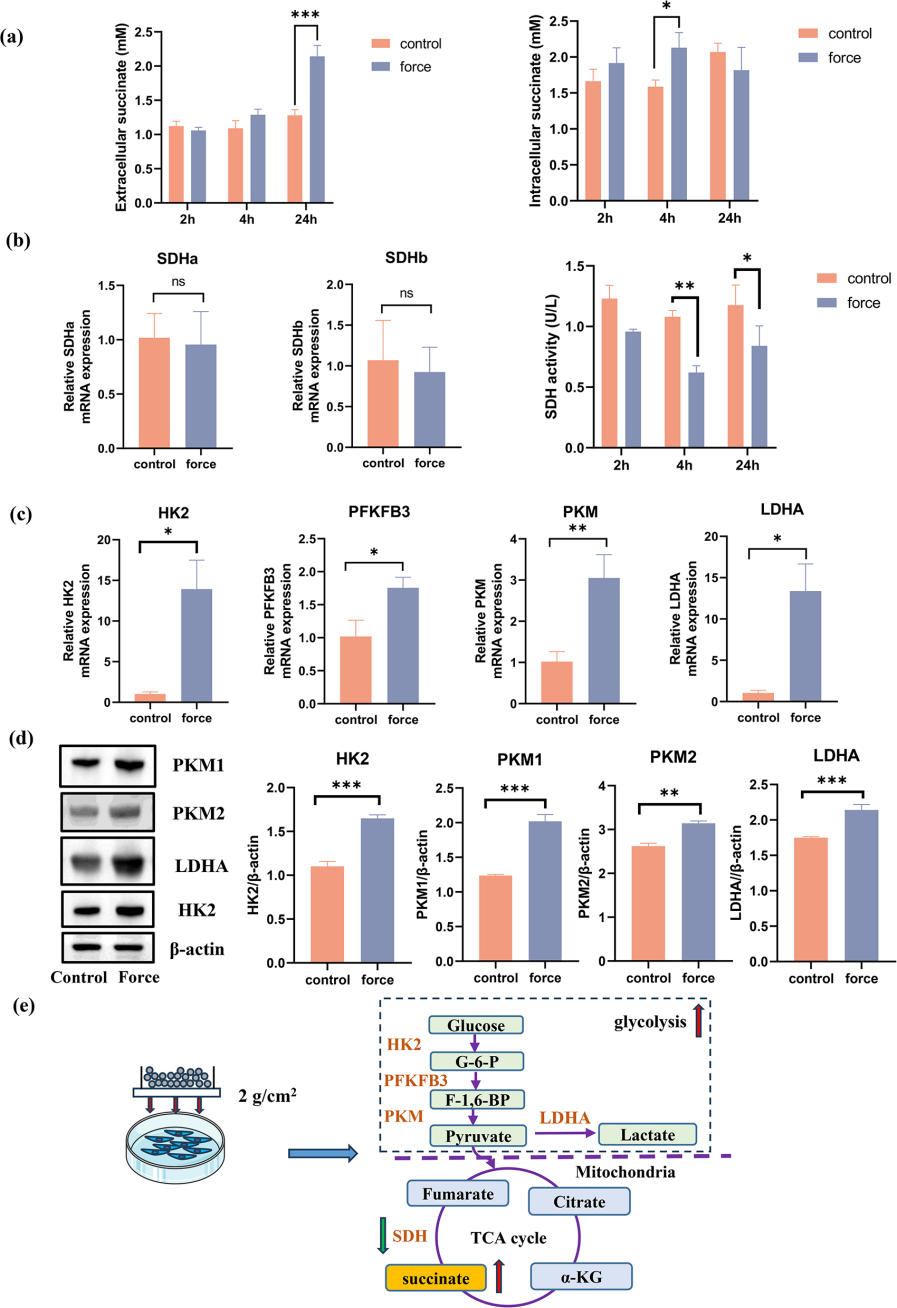
Compressive force induced succinate production in PDLCs with increase of glycolysis and decrease of SDH activity. **a** Levels of succinate in cell supernatant and cell lysates were tested by the succinate colorimetric assay. **b** The transcription level of succinate dehydrogenase (SDH) a and b were analyzed by qPCR (2 h) and the enzymatic activity of SDH decreased in PDLCs after stimulated with mechanical force for 4 and 24 h. **c** The transcription level of hexokinase 2 (HK2), 6-phosphofructo-2-kinase/fructose-2,6-biphosphatase 3 (PFKFB3), pyruvate kinase (PKM) and lactate dehydrogenase A (LDHA) increased in force-treated PDLCs. **d** The protein expression of PKM1, PKM2, LDHA and HK2 was measured in force-treated PDLCs. The semi-quantitative analysis was performed with Image-J. **e** Schematic diagram of changes in force-stimulated PDLCs. Succinate accumulated with the increase of glycolysis and the decrease of SDH enzymatic activity. *p < 0.05; ** *p* < 0.01; *** *p* < 0.001

PDLCs were seeded into six-well plates for 24 h and subsequently treated with continuous compressive force (Fig. 2e). Specifically, a glass slide was placed on the PDLCs layer in the well and the force was adjusted by adding or removing steel balls in the glass bottle. PDLCs were subjected to a compressive force of 2.0 g/cm^2^. In the control group, PDLCs were cultured without compressive force.

#### RNA extraction and real-time polymerase chain reaction

Total RNA was extracted using SteadyPure Universal RNA Extraction Kit (AG21024, Accurate Biotechnology, Hunan, China). Reverse transcription to complementary DNA was performed using the *EVO M-MLV* RT Premix for qPCR (AG11706, Accurate Biotechnology, Hunan, China) according to the manufacturer’s protocol. Then, the quantification of the relative transcription level, using SYBR^®^ Premix (AG11718, Accurate Biotechnology, Hunan, China), was normalized to the β-actin transcription level. The primer sequences were listed in Table 1.

**Table 1:** Sequences of the primers

Primers	Forward Primer (5′ to 3′)	Reverse Primer (3′ to 5′)
Human		
PFKFB3	CACGGCGAGAATGAGTACAA	TTCAGCTGACTGGTCCACAC
PKM	CCGCCTGGACATTGATTCAC	GTGTCTAGAGCCACAGCAAC
LDHA	AGCTTCCATTTAAGGCCCCG	TCTTTTGAGACCGCTAGTGC
HK2	GAGCCACCACTCACCCTACT	CCAGGCATTCGGCAATGTG
SDHa	CAGCATGTGTTACCAAGCTGT	GGTGTCGTAGAAATGCCACCT
SDHb	ACCTTCCGAAGATCATGCAGA	GTGCAAGCTAGAGTGTTGCCT
ACTIN	GTGGGGCGCCCCAGGCACCA	CGGTTGGCCTTGGGGTTCAGGGGGG
Mouse		
NFATc1	GACCCGGAGTTCGACTTCG	TGACACTAGGGGACACATAACTG
CTSK	GACCCGGAGTTCGACTTCG	TGACACTAGGGGACACATAACTG
MMP9	GAAGAAGACTCACCAGAAGCAG	TCCAGGTTATGGGCAGAGATT
RANKL	AGCCGAGACTACGGCAAGTA	AAAGTACAGGAACAGAGCGATG
OPG	ACCCAGAAACTGGTCATCAGC	CTGCAATACACACACTCATCACT
TRAP	CACTCCCACCCTGAGATTTGT	CCCCAGAGACATGATGAAGTCA
SUCNR1	GGAGACCCCAACTACAACCTC	AGCAACCTGCCTATTCCTCTG
ACTIN	GCCAACCGTGAAAAGATGACC	GAGGCATACAGGGACAGCAC

PFKFB3, 6-phosphofructo-2-kinase/fructose-2,6-biphosphatase 3; PKM, Pyruvate Kinase; LDHA, Lactate dehydrogenase A; HK2, Hexokinase 2; SDHa, Succinate Dehydrogenase a; SDHb, Succinate Dehydrogenase b; NFATc1, Nuclear factor of activated T-cells, cytoplasmic 1; CTSK, Cathepsin K; MMP9, matrix metalloprotein 9; RANKL, receptor activator of nuclear factor kappa-B ligand; OPG, Osteoprotegrin; TRAP, tartrate resistant acid phosphatase; SUCNR1, Succinate receptor 1

#### Measurement of succinate concentration and succinate dehydrogenase activity

The concentration of extracellular and intracellular succinate was measured using a succinate assay kit (K-SUCC, Megazyme, Ireland) according to the manufacturer’s instruction. For extracellular succinate measurement, the cell culture supernatant was collected. At the same time, the cells were subjected to a freeze-thaw cycle to collect cell lysates for intracellular succinate measurement. Afterwards, the solution of cell lysis was centrifuged at 1,200 rpm for 5 min to remove cellular debris. Succinate dehydrogenase (SDH) activity was detected with a SDH activity assay kit (E-BC-K649-M, Elabscience Biotechnology, China) according to the manufacturer’s protocol.

#### Wound healing migration assay

A straight scratch was made using a 200 µL pipette tip. After rinsing with PBS for 3 times, the cells were incubated with serum-free DMEM with different concentration of succinate. Images were captured at 0 and 24 h under 4× magnification. Subsequently, the images were further analyzed by ImageJ software to quantify the wound closure percent at the same reference point.

#### Transwell assay

A total of 100 µL suspension (cell density adjusted to 2 × 10^5^ cells/mL) with or without succinate weas loaded into the upper compartment of 24-well transwell culture chamber with a 8-µm pore size (Corning, USA). Meanwhile, 600 µL medium containing 10% fetal bovine serum was added to the lower compartment. After incubation at 37◦C for 3 days, the migrated cells were fixed with 4% paraformaldehyde for 15 min and stained with 0.2% crystal violet (C0121, Beyotime, China) for 10 min. The number of migrated cells in three randomly selected fields was counted under a microscope.

#### Tartrate-resistant acid phosphatase (TRAP) staining for the detection of osteoclasts

BMDMs were cultured in the aforementioned osteoclast induction medium with or without the addition of succinate (5 mM) for 7 days. Subsequently, the cells were rinsed three times with PBS and fixed with 4% paraformaldehyde for 10–15 min. Tartrate-resistant acid phosphatase (TRAP) staining (G1492, Sariobio, China) was performed in a dark environment for 60 min, followed by counterstaining of the nuclei with hematoxylin (G10005, Servicebio, China) for 1–2 min. After rinsing three times with PBS, the samples were observed under an optical microscope.

### Western blot analysis

Cells were washed with PBS and lysed with RIPA lysis buffer for 10 min on ice. The concentration of total proteins was determined using a Nanodrop spectrophotometer (Thermo Fisher, USA). Equal amounts of protein were separated via 4–12% SDS-PAGE (M00938, Genscript, China) and transferred to a PVDF membrane. The membranes were blocked with 5% bovine serum albumin (Sigma, USA) and incubated overnight at 4 °C with primary antibodies against PKM1(D30G6,CST, USA), PKM2 (D78A4, CST, USA), LDHA (19987-1-AP, ProteinTech, China), HK2(ab209847, Abcam, USA), SUCNR1 (AF5316, Affinity Biosciences, USA), Matrix Metalloproteinase 9 (MMP9, A0289, ABclonal, China), TRAP (EPR15556, Abcam, USA) or β-actin (GB15003-100, ServiceBio, China). Membranes were then incubated with secondary antibodies (GB23303, ServiceBio, China) at room temperature for 1 h. Finally, the membranes were exposed to an ECL reagent (P10060, NCM Biotech, China) for signal detection. Images were captured using a Tanon 6200 Luminescent Imaging Workstation (Tanon, China).

### In vivo animal experimental protocols

#### OTM model establishment

All experiments involving mice were carried out under the approval of the Committee on the Ethics of Animal Experiments of Nanjing University (IACUC- D2202111). Healthy male C57BL/6 mice and SUCNR1 knockout mice (SUCNR1-/-) (GemPharmatech, Nanjing, China) aged 6–8 weeks were used in this study (*n* = 8 per group).

Air temperature and humidity for mice were controlled at 22 °C and 40–60%, respectively.

A 0.20-mm Ni-Ti spring (American Orthodontic, USA) was fixed between the first right molar and incisor of the upper jaw in mice, applying 20 g stretch force. The spring in the anterior tooth area was secured with light-curing resin. Mice were checked daily to ensure the spring remained in place, and they were fed with water and soft food. The mice were sacrificed on day 7. The maxilla was divided into left and right parts, with the left side serving as the control group. Samples were fixed in 4% paraformaldehyde for subsequent experiments.

#### Micro-CT and TRAP staining

Samples were scanned with a micro-CT imaging system (Skyscan 1076; Bruker, Kontich, Belgium). Three-dimensional images were reconstructed using CTvox and Date Viewer software. The shortest distance between the crowns of first and second molars was defined as the OTM distance. TRAP staining was performed following the manufacturer’s protocol (G1492, Sariobio, China).

### Statistical analysis

Statistical and pathway analysis of GC–MS data were conducted with MetaboAnalyst ([22], version 5.0) to analyze differential metabolites and pathways during OTM. A two-tailed T-test was conducted to compare differences between different time points. Differential metabolites were defined based on criteria of *p-*values < 0.05 and variable importance in projection (VIP) > 1.0. The Kyoto Encyclopedia of Genes and Genomes (KEGG) database and the Human Metabolome Database (HMDB) were utilized to evaluate genes related to differentially-expressed metabolites.

Other Data were presented as mean ± standard deviation (SD) and analyzed with GraphPad Prism 8. Comparisons between the two groups were conducted with Student’s t-tests or Welch t-tests, depending on the homogeneity test of variance. One-way analysis of variance (ANOVA) was conducted for comparisons between multiple groups. For multiple related samples, the Friedman test was applied. *p* < 0.05 was identified as statistical difference.

## Results

### Succinate production increased in GCF during the progression of OTM

A total of 13 subjects were enrolled and 52 samples were tested in this study. The age, sex, PD, CAL and crowding degree between the mandibular canines were recorded at the onset of orthodontic treatment (Table 2). All subjects were female, with a mean age of 24.2 ± 2.3 years (range: 20–28 years). The mean value of crowding was 5.8 ± 1.1 mm before orthodontic treatment. The plaque index and gingival index at different time points were presented in Table 3. A slight increase in PLI and GI was observed, but no significant difference was detected over time.

**Table 2:** Demographic and clinical characteristics of the subjects at the initial of orthodontic treatment (mean ± SD/n)

Clinical parameters	T0
Number of subjects	13
Age (range)	24.2 ± 2.3 (20–28)
Sex (Male/Female)	0/13
PD (mm)	1.9 ± 0.6
CAL (mm)	0
crowding degree (mm)	5.8 ± 1.1

CAL, clinical attachment level; PD, probing depth

**Table 3:** The PLI and GI of mandibular anterior teeth in all subjects at different timepoints (mean ± SD)

Timepoint	PLI	GI
T0	1.27 ± 0.64	1.18 ± 0.64
T1	1.31 ± 0.61	1.22 ± 0.64
T2	1.50 ± 0.64	1.23 ± 0.68
T3	1.51 ± 0.62	1.26 ± 0.69

PLI, plaque index; GI, gingival index

Multivariate evaluations between different time points were performed with Principal Component Analysis (PCA) and Partial Least Squares Discriminant Analysis (PLS- DA) model. A total of 79 metabolites were detected in the GCF. This study included sampling at 0, 1, 7 and 28 days post-orthodontic treatment to observe the dynamic metabolic changes during the procedure of OTM. The PLS-DA analysis based on two principal components revealed a distinct separation in the metabolic profiles at different time points, particularly at 7 days post-orthodontic treatment (T2) (Fig. 1b).

The combination of VIP and *p*-value was employed to identify differential metabolites. At 7 days post-orthodontic treatment (T2), six elevated differential metabolites were detected in GCF compared with T0 (Fig. 1c). The six differential metabolites identified were cholesterol, succinic acid, lauric acid, serine, behenic acid and cytidine-5’-diphosphate (VIP > 1.0). At 28 days post-orthodontic treatment (T3), a reduction of maltotriose and an increase of serine were observed in GCF compared with T0, with VIP values > 2.5 (Fig. 1d). The pathway analysis illustrated that nine pathways were significantly different between T0 and T2, including butanoate metabolism, citrate cycle (TCA cycle), propanoate metabolism, and alanine, aspartate and glutamate metabolism (Fig. 1e). An elevated level of succinate was observed at 7 days post-orthodontic treatment. Meanwhile, pathways associated with the TCA cycle were significantly different at the same time.

### Compressive force induced succinate production via the increase in Glycolysis and the decrease in SDH activity in PDLCs

The concentration of succinate was differentially increased in the force-treated group compared with the control group in PDLCs (Fig. 2a). To further explore the cause of the elevated succinate, the expression levels of enzymes related to the TCA cycle and glycolysis were measured. Although the mRNA expression levels of the genes encoding the SDH subunits SDHa and SDHb remained unchanged after 2 h of compressive force treatment, the enzymatic activity of SDH in PDLCs was significantly reduced at 4 h and 24 h following compressive force treatment (Fig. 2b). The mRNA expression of glycolysis-related enzymes, such as HK2, PFKFB3, PKM and LDHA, increased at 2 h after compressive force treatment (Fig. 2c). Moreover, the protein levels of these enzymes significantly increased at 24 h (Fig. 2d). Schematic diagram of changes in compressive force-stimulated PDLCs was shown in Fig. 2e.

### Succinate promoted the migration of PDLCs and the recruitment of macrophages

**Fig. 3:**
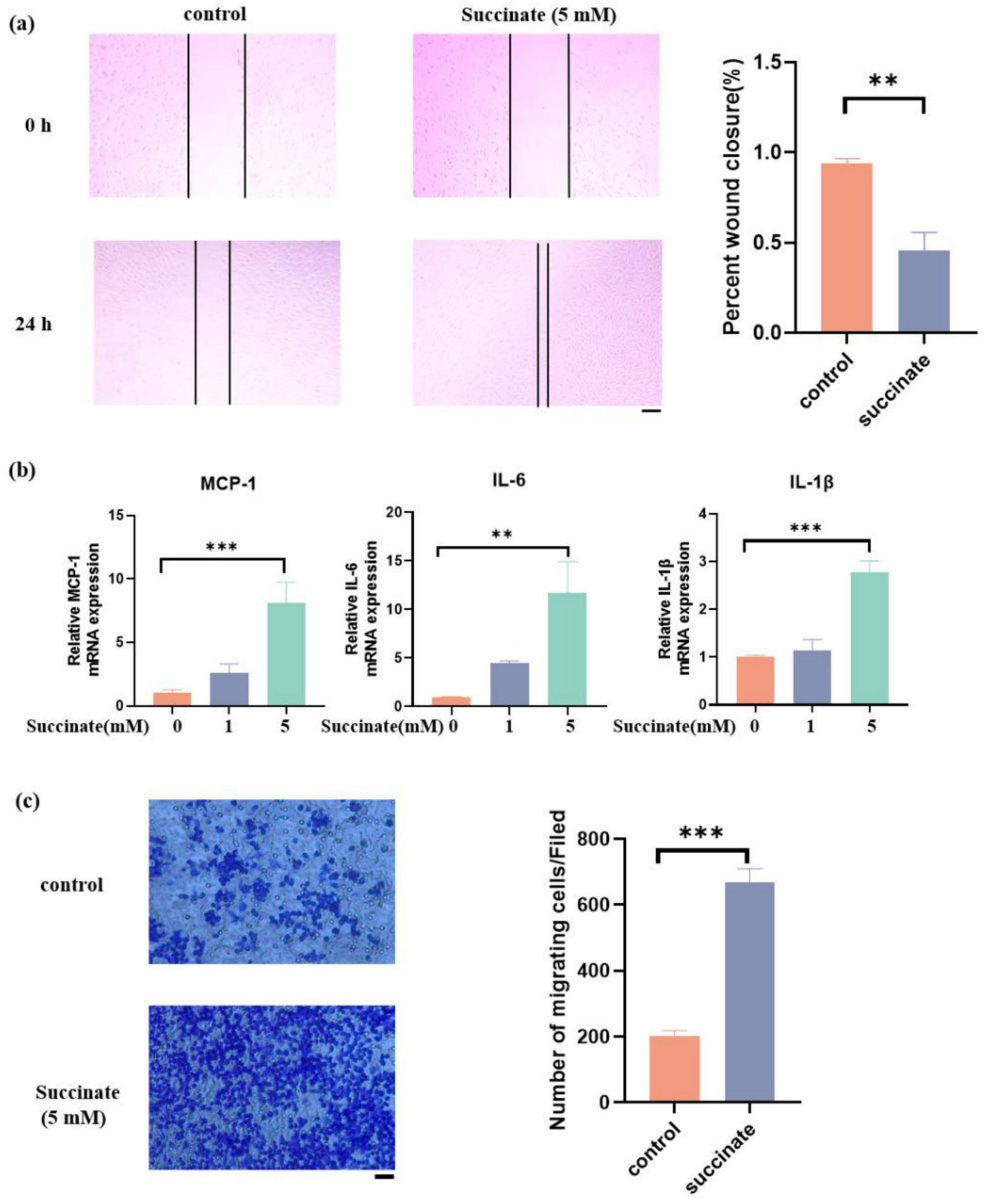
Succinate promoted migration of PDLCs and caused local aseptic inflammation with the recruitment of macrophages. **a** Representative images of wound-healing assay after building scratch-healing model for 0 h and 24 h to determine the migration of PDLCs (Scale bar: 200 μm). The percent of wound closure was calculated. **b** The expression of inflammatory cytokines genes in PDLCs after stimulation with different concentrations of succinate for 4 h. **c** Transwell migration assay of bone marrow monocytes (BMDMs). (Scale bar: 100 μm). *p < 0.05; ** *p* < 0.01; *** *p* < 0.001

Succinate enhanced the migration of PDLCs (Fig. 3a). Furthermore, succinate upregulated the expression levels of monocyte chemotactic protein-1 (MCP-1), Interleukin-1β (IL-1β) and Interleukin-6 (IL-6) (Fig. 3b). For macrophages, during OTM in vivo, metabolic products produced by PDLCs are continuously and slowly released into the surrounding microenvironment, and macrophages are stimulated by these metabolic products over a relatively long period of time. To explore whether succinate derived from PDLCs acted as a chemokine for macrophages, the migration of BMDMs was measured with a Transwell assay. The results showed that succinate dramatically enhanced the migration of BMDMs (Fig. 3c).

### Blockade of SUCNR1 or SUCNR1 deficiency impeded osteoclastogenesis to decrease OTM

**Fig. 4:**
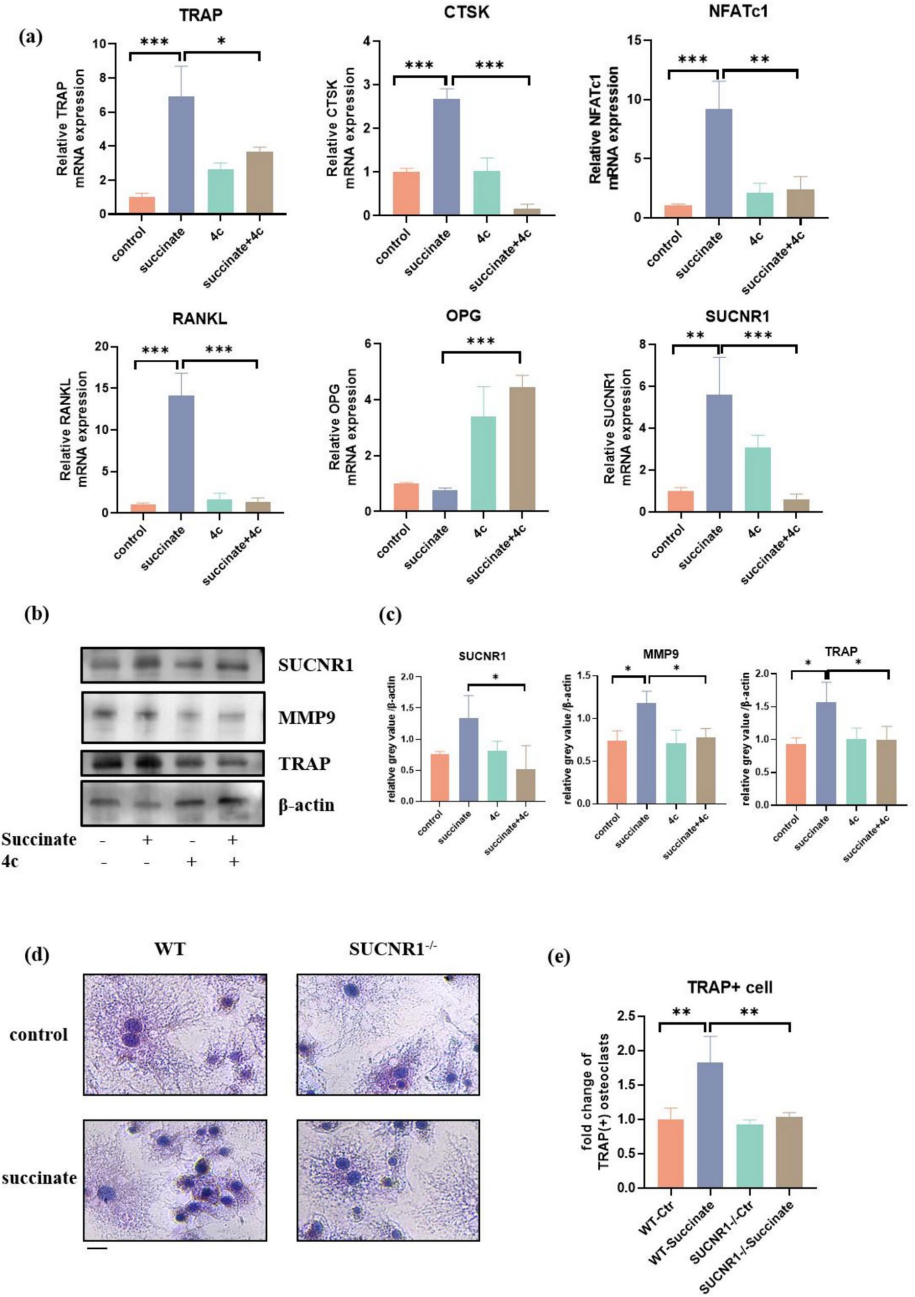
Blockage of SUCNR1 impedes osteoclastogenesis to alleviate OTM. BMDMs were harvested from SUCNR1-/- and WT mice. BMDMs were cultured with 30 ng/ml M-CSF and 50 ng/mL RANKL to induce osteoclast formation, and 5 mM succinate or 10 µM 4c was added to the culture medium. **a** mRNA of TRAP, CTSK, NFATc1, RANKL and SUCNR1 was measured on day 3 by qPCR. **b** Protein level of osteoclast markers (TRAP and MMP9) and SUCNR1was measured by Western blot on day 5. **c** The protein level of SUCNR1, MMP9 and TRAP was upregulated in the succinate-treated group, which could partly be reversed by 4c. **d**, **e** Tartrate-resistant acid phosphatase (TRAP) staining was used to stain osteoclasts after succinate (5 mM) stimulation. Scale bar: 100 μm

To further investigate the effects of succinate-SUCNR1 axis in BMDMs, we applied the SUCNR1 antagonist 4c to inhibit the function of succinate. The mRNA and protein expression levels of SUCNR1 were upregulated by exogenous succinate and inhibited by 4c (Fig. 4a-b). The increased expression of osteoclastic-related gene markers (TRAP, CTSK, NFATc1 and RANKL) induced by exogenous succinate was remarkably suppressed by 4c at both the gene (Fig. 4a) and protein levels (Fig. 4b-c). Consistently, TRAP staining showed that the number of TRAP (+) cells increased after treatment with succinate and blockade of SUCNR1 decreased the number of TRAP (+) cells (Fig. 4d-e).

**Fig. 5:**
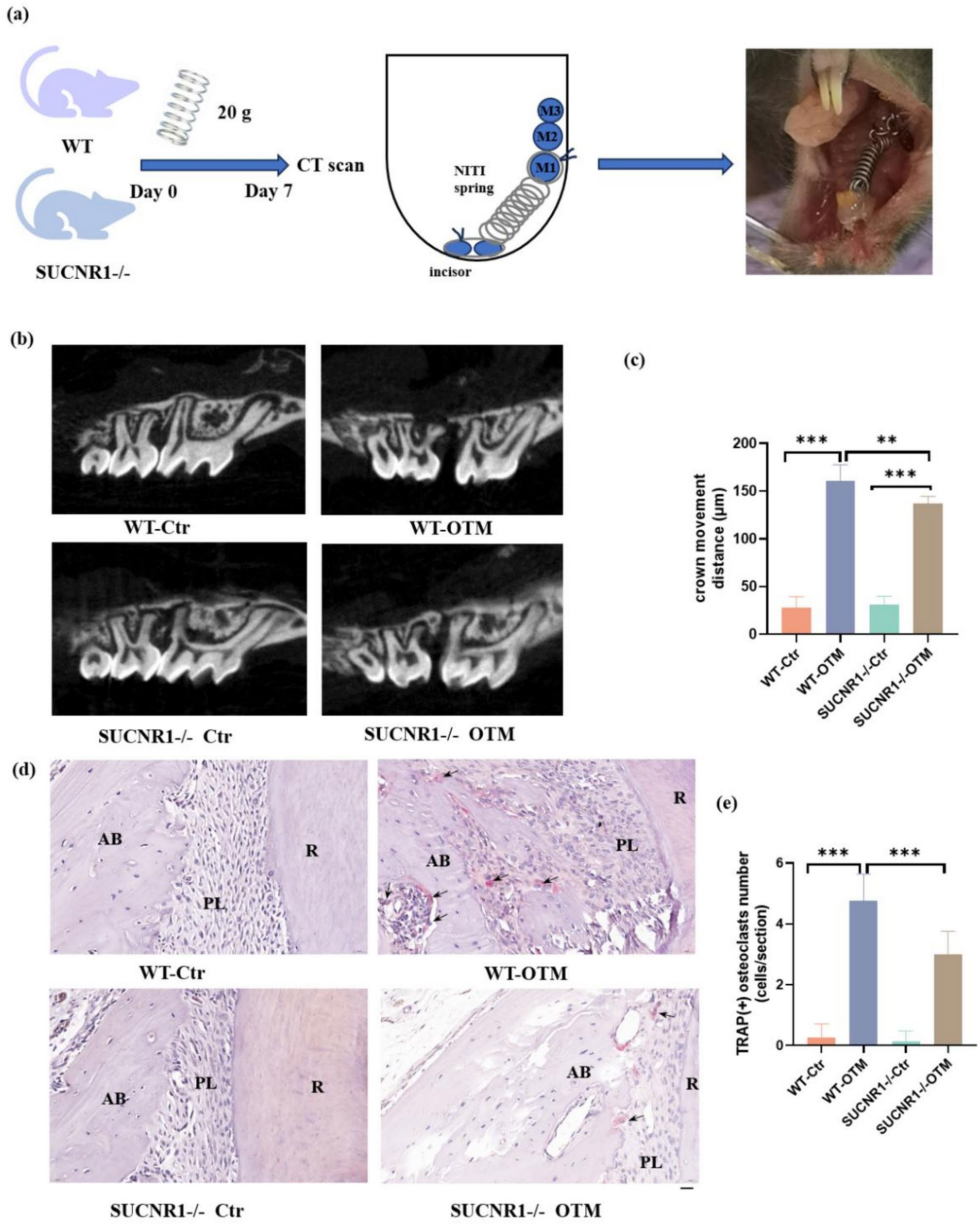
SUCNR1 deficiency decreased OTM. OTM model was established in both SUCNR1-/- and WT mice. **a** Schedule diagram and intraoral photograph of in vivo experiments. **b**, **c** The micro-computed tomography (micro-CT) views showed the crown movement distance of the maxillary first molars at 7 days after orthodontic force application. **d** Tartrate-resistant acid phosphatase (TRAP) staining was used to stain osteoclasts in the alveolar bone on the pressure side of M1 roots. The arrows indicated the osteoclasts. **e** In OTM group, SUCNR1-/- mice represented a considerable decrease in osteoclast numbers in bone tissue areas compared to the WT mice. (Scale bar: 20 μm. M1, the first maxillary molar; AB, alveolar bone; R, root.) *p < 0.05; ** *p* < 0.01; ***p < 0.001

To directly demonstrate the function of SUCNR1 in OTM, we established an OTM model in SUCNR1-/- and WT mice (Fig. 5a). 7 days after the application of orthodontic force, SUCNR1-/- mice exhibited significantly shorter movement distance than the WT mice (Fig. 5b-c). Furthermore, SUCNR1-/- mice subjected to orthodontic force represented a considerable decrease in osteoclast numbers in bone tissue areas on pressure side (Fig. 5d-e) compared to the WT mice.

## Discussion

OTM, an adaptation of the periodontal tissue to the orthodontic force, is accompanied by dynamic metabolic changes in the periodontium to fulfil the alveolar bone remodeling. In addition to the release of pro-inflammatory mediators such as prostaglandin E2, IL-1β, and tumor necrosis factor α in GCF [25], our study focus on the changes of metabolic substances during OTM for the first time. The result identifies succinate as a critical factor in OTM-related bone resorption, enhancing our understanding of the metabolic processes involved in OTM.

Under the compressive stimulation, PDLCs undergo metabolic reprogramming, leading to the accumulation of succinate. The succinate can be transported into the cytosol and extracellular space; therefore, succinate can be a metabolite that transmit the metabolic signals in the mitochondria [26]. The accumulation of succinate might be a result of inhibited activity of succinate dehydrogenase [13] and the activation of glycolysis; thus, our result showed that TCA cycle was dampened in GCFs in the stage of OTM or compressive force treated PDLCs.

Compressive force leads to local microvessel obstruction of on the pressure side and may impair cell function. In the tensile region, intact PDLCs migrate under the stimulation of succinate, and this process may promote tissue repair and tooth movement. Meanwhile, intracellular succinate not only amplifies the inflammatory response within PDLCs but also recruits macrophages. Succinate released by PDLCs binds to SUCNR1 on macrophages, leading to osteoclast-related gene activation and bone resorption on the pressure side. The activation of SUCNR1 has been confirmed to be related to metabolic stress in pathological or physiological state especially ischemia/hypoxia [27]. Therefore, SUCNR1 is a physiological sensor sensitive to the elevation of succinate and succinate-SUCNR1 acts role in the transcription of immune function genes in macrophages [17].

In the periodontal milieu, succinate supplement activates SUCNR1 to exacerbate experimental periodontitis via exaggerated periodontal inflammatory responses [28]. Meanwhile, in hyperglycaemic conditions, suppression of the receptor with SUCNR1 antagonist 4c effectively inhibits osteoclast differentiation and bone destruction [29]. Our present study first demonstrated that succinate-SUCNR1 axis participated in the process of OTM in the stage of alignment, offering potential for clinical applications. However, succinate induced the production of pro-inflammatory cytokines and was confirmed to be related with sustained inflammatory response [5, 30]. It must be emphasized that excessive succinate may cause persistent inflammation, raising the risk of bone destruction and orthodontically induced inflammatory root resorption (OIIRR) [24, 31]. Therefore, an appropriate amount of succinate restoring metabolic stability were required during the process of OTM.

Several issues should be noted for our present study. Firstly, the collection sites of GCF were not separated into pressure and tension sides. Since the gingival sulcus around a tooth acted as a single reservoir for GCF, metabolite variations may be balanced and masked [32]. Secondly, the number of experimental samples, including clinical specimens and animal models, was determined based on previous related studies rather than rigorous statistical calculations. Thirdly, the whole set of PDLCs, osteoblasts, osteoclasts and osteocytes participate in the alveolar bone remodeling during OTM. PDLCs produce RANKL under orthodontic forces; however, the alveolar bone resorption process is majorly finely tuned by the couple of the osteoblasts and osteoclasts, chiefly through the balance of RANKL/OPG. Future research should focus on the crosstalk between different cells in periodontal ligament. In addition, in the WT group, groups with local injection of succinate, as well as groups with orthodontic force combined with succinate injection, should be set up. Additionally, further investigation is necessary to verify the safety and systemic effects of succinate.

**Fig. 6:**
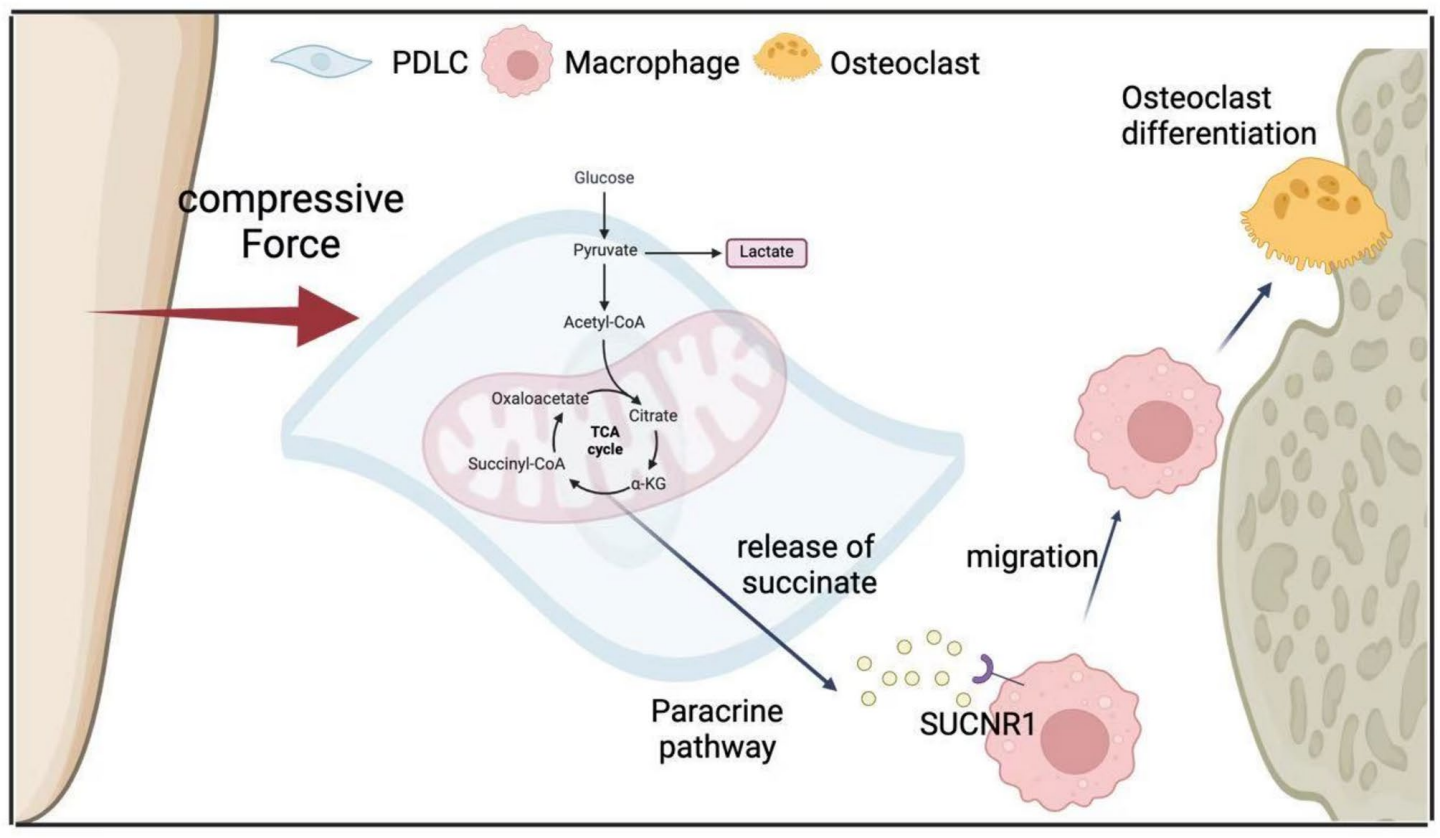
Schematic illustration: compressive force-induced succinate production via metabolic reprogramming in periodontal ligament cells promoting osteoclastogenesis in orthodontic tooth movement

In summary, compressive force led to an increase in glycolysis and hindered the tricarboxylic acid (TCA) cycle in periodontal ligament cells, which in turn results in the accumulation of succinate. Moreover, succinate further stimulates the migration of macrophages and their osteoclastic differentiation. Application of succinate and targeting SUCNR1 may be utilized to control OTM through regulating osteoclast differentiation (Fig. 6).

## Conclusion

This study reveals that compressive force during OTM induces metabolic reprogramming in PDLCs, increasing succinate production. The succinate-SUCNR1 axis promotes macrophage-mediated osteoclastogenesis and bone resorption, which are essential for tooth movement. These findings enhance our understanding of orthodontic mechanisms and suggest a new therapeutic target for orthodontic treatment optimization.

## Data Availability

The datasets used and/or analysed during the current study are available from the corresponding author on reasonable request.

## Abbreviations

OTM: Orthodontic tooth movement
PDLCs: Periodontal ligament cells
BMDMs: Bone marrow-derived macrophages
SUCNR1: Succinate receptor 1
PFKFB3: 6-Phosphofructo-2-kinase/fructose-2,6-biphosphatase 3
PKM: Pyruvate kinase
LDHA: Lactate dehydrogenase A
HK2: Hexokinase 2
SDHa: Succinate dehydrogenase a
SDHb: Succinate dehydrogenase b
NFATc1: Nuclear factor of activated T-cells, cytoplasmic 1
CTSK: Cathepsin K
MMP9: Matrix metalloprotein 9
RANKL: Receptor activator of nuclear factor kappa-B ligand
OPG: Osteoprotegrin
Micro-CT: Micro-computed tomography
TRAP: Tartrate resistant acid phosphatase
CAL: Clinical attachment level
PD: Probing depth
PLI: Plaque index
GI: Gingival index
